# Editorial: Herbal medicines and their metabolites: effects on lipid metabolic disorders via modulating oxidative stress

**DOI:** 10.3389/fphar.2023.1279429

**Published:** 2023-09-15

**Authors:** Yunhui Chen, Yongmei Xie, Cailu Lin, Wei Peng

**Affiliations:** ^1^ School of Basic Medical Sciences, CDUTCM-KEELE Joint Health and Medical Sciences Institute, School of Pharmacy, Chengdu University of Traditional Chinese Medicine, Chengdu, China; ^2^ State Key Laboratory of Biotherapy, West China Hospital, Sichuan University, Chengdu, China; ^3^ Monell Chemical Senses Center, Philadelphia, United States

**Keywords:** herbal medicines, natural metabolites, oxidative stress, lipids, lipid metabolic disorders

Lipids, predominantly triglycerides, phospholipids, and sterols, play pivotal roles in human physiology, particularly nutrient regulation. However, imbalances in lipid metabolism may precipitate a range of severe health conditions, such as non-alcoholic fatty liver diseases, hyperlipidemia, diabetes mellitus, and atherosclerosis. An emerging concern is oxidative stress, a detrimental state arising from an imbalance between oxidation and antioxidation, primarily due to the surge in reactive oxygen species. Mounting evidence associates oxidative stress and mitochondrial dysfunction with lipid metabolic disorders. Intriguingly, recent studies have spotlighted the potential of herbal medicines and their metabolites in modulating oxidative stress and countering these disorders. This Research Topic, “*Herbal Medicines and Their Metabolites: Effects on Lipid Metabolic Disorders via Modulating Oxidative Stress*”, has provided an academic platform for scholars to delve into the potential of various herbal interventions in addressing metabolic challenges, and 11 good quality works are collected in our Research Topic.

The original research by Zhang et al. explored the antidiabetic effects of hydroxy-α-sanshool (HAS) from *Zanthoxylum bungeanum* Maxim on streptozotocin-induced Type 2 diabetes mellitus (T2DM) mice and glucosamine (GlcN)-induced HepG2 cells, and the results indicated its mechanism of actions might attribute to the increase of hepatic glycogen synthesis by activating PI3K/Akt/GSK-3β/GS signaling pathway. Another experimental research by Cen et al. reported the ameliorative effects of anti-malarial artesunate, a succinate derivative of artemisinin, on atherogenic diet and lipopolysaccharide-induced atherosclerosis rat by mitigating arterial inflammatory responses via the inhibition of the NF-κB-NLRP3 inflammasome pathway.

Furthermore, a series of insightful and valuable reviews have been included to present the recent advances in the field. He et al. comprehensively summarized natural polysaccharides with promising therapeutic efficacy against T2DM and elaborated on their pharmacological mechanisms relevant to the oxidative stress network. Li et al. illustrated sixteen natural flavonoids as potential anti-atherogenic agents that inhibited oxidative stress in endothelial cells, accentuating the importance of further research on quercetin and naringenin. Sheng et al. further enriched the preventive and therapeutic roles of catechins, a major group of flavonoids, against atherosclerosis via regulating antioxidant stress and improving abnormal lipid metabolism. Duan et al. reviewed the herbal medicines against low-density lipoprotein (LDL) oxidation and foam cell formation in lipid metabolism disorder, highlighting that herbal interventions could inhibit LDL oxidation and regulate cholesterol homeostasis via downregulating CD36 and SR-A, whereas upregulating ABCA1 and ABCG1. Luo et al. reported that botanical drugs could regulate radical oxygen species via multiple signaling pathways to improve oxidative stress and manage glucolipid metabolic diseases. Jin et al. elucidated the multifaceted molecular mechanisms through which ginsenosides regulate oxidative stress and lower blood lipids in treating hyperlipidemia and its associated diseases.

In addition, Xie et al. presented meta-analyses of animal studies to summarize the hypoglycemic effects of ginsenoside rg1 and provided evidence-based support for its efficacy in reducing MDA levels and restoring SOD activity to exert its antioxidant activity and had a positive effect on the reduction of IL-6 and TNF-α levels. Li et al. also conducted a meta-analysis to provide preclinical and clinical evidence for the treatment of NAFLD with soybean, and the results verified that soybean could protect the liver in NAFLD by regulating lipid metabolism and oxidative stress factors via the Akt/AMPK/PPARα signaling pathway. Moreover, an empirical study by Dai et al. testified that no substantial difference was noted in the effects between randomized controlled trials (RCTs) and non-randomized studies of interventions (NRSIs) in safety assessment when they have similar sample sizes, indicating evidence from NRSIs might be considered a supplement to RCTs for safety outcomes.

This research compendium has illuminated the profound potential of modulating oxidative stress with herbal medicines in treating various lipid metabolic disorders. These *in vivo* and *in vitro* experiments, reviews, and meta-analyses collectively accentuate the promise of targeting oxidative stress with herbal remedies against health challenges of lipid dysmetabolism. Nevertheless, several facets remain uncharted as we delve deeper into this area. More original research is warranted in the future to explore novel pharmacological insights into herbal medicines for managing lipid metabolic disorders; elucidate the intricate molecular mechanisms underlying the therapeutic effects of herbal medicines; identify herbal medicines that may potentially mitigate the side effects of contemporary lipid metabolic disorder drugs; and integrate such strategies as network pharmacology, artificial intelligence, and computer-aided design to discover herbal interventions targeting oxidative stress.

